# RISK FACTORS FOR RENAL CALCULI IN PATIENTS WITH CROHN’S DISEASE

**DOI:** 10.1590/S0004-2803.24612024-023

**Published:** 2025-05-02

**Authors:** Dídia Bismara CURY, Liana Cristina Bismara CURY, Ana Camila MICHELLETTI, Rogerio Antonio OLIVEIRA, Elsa Alidia Petry GONÇALVES, Nestor SCHOR

**Affiliations:** 1 Clínica Scope, Centro de Doenças Inflamatórias Intestinais, Campo Grande, MS, Brasil.; 2 Universidade UNIDERP Anhanguera, Clínica Scope, Centro de Doenças Inflamatórias Intestinais, Campo Grande, MS, Brasil.; 3 Universidade Federal de Mato Grosso do Sul, Instituto de Química, Campo Grande, MS, Brasil.; 4 Universidade Estadual Paulista Julio de Mesquita Filho, Departamento de Bioestatística, Botucatu, SP, Brasil.; 5 Universidade Federal de São Paulo, Divisão de Nefrologia, Departamento de Medicina, São Paulo, SP, Brasil.; 6 Universidade Federal de Mato Grosso do Sul, Campo Grande, MS, Brasil.; 7 Departamento de Nefrologia, Universidade Federal de São Paulo, São Paulo, SP, Brasil.

**Keywords:** Kidney stones, nephrolithiasis, Crohn’s disease, risk factor, Pedras nos rins, nefrolitíase, doença de Crohn, fator de risco

## Abstract

**Background:**

The incidence of nephrolithiasis has increased significantly, diet, obesity, and high animal protein intake having been reported to be risk factors. Nephrolithiasis has a great economic impact on society related to work absenteeism, recurrent attacks of renal colic, and urological interventions. Nephrolithiasis can also progress to renal failure. It is therefore important to identify the risk factors for nephrolithiasis. Inflammatory bowel diseases, which include Crohn’s disease, represent a risk factor for the formation of renal calculi, due to the disease itself and to the use of drugs that can influence the metabolism of substances related to nephrolithiasis. In the past, Crohn’s disease patients were often submitted to surgery, which is known to contribute to nephrolithiasis. New drugs have changed the clinical course of Crohn’s disease, whose incidence has increased worldwide. Specialists should be on the alert not only for the complications of Crohn’s disease but also for its extraintestinal manifestations, which can dramatically affect the quality of life of these patients and lead to renal failure. It is therefore important to screen this population for nephrolithiasis.

**Objective:**

To determine the prevalence of nephrolithiasis in a population of patients with Crohn’s disease; to determine whether drugs, previous surgery, location of the disease, and clinical activity are risk factors for nephrolithiasis; and to alert specialists to the importance of screening for nephrolithiasis (through simple methods) in order to prevent chronic kidney disease.

**Methods:**

Were analyzed the electronic medical records of 93 Crohn’s disease patients treated between 2009 and 2010 at the Inflammatory Bowel Disease Center of the Scope Clinic, located in the city of Campo Grande. All of the patients underwent ultrasound at the first medical appointment.

**Results:**

Of the 93 patients, 37 developed nephrolithiasis at some point during the study period. Risk factors for nephrolithiasis were disease location (*P*=0.023) and the use of ciprofloxacin (*P*=0.0001), corticosteroids (*P*=0.005), immunomodulators (*P*=0.001), or metronidazole (*P*=0.0005). Surgical status, age, and gender were not found to predispose to the formation of renal calculi.

**Conclusion:**

This study demonstrates the importance of using imaging methods to screen Crohn’s disease patients for nephrolithiasis, regardless of their surgical status.

## HIGHLIGHTS

• Patients with inflammatory bowel diseases, especially Crohn’s disease, may be more likely to develop kidney stones (nephrolithiasis).

• The incidence of nephrolithiasis has increased in recent years.

• The location of the disease and the drugs that are used for the treatment patients with Crohn’s disease constitute major risk factors for developing nephrolithiasis.

• This study reveals that using imaging methods to screen Crohn’s disease patients for nephrolithiasis it is a great medical tool.

## RESUMO

**Contexto:**

A incidência de nefrolitíase aumentou significativamente, tendo sido relatado que dieta, obesidade e alta ingestão de proteína animal são fatores de risco. A nefrolitíase tem um grande impacto econômico na sociedade relacionado com o absenteísmo laboral, crises recorrentes de cólica renal e intervenções urológicas. A nefrolitíase também pode evoluir para insuficiência renal. Portanto, é importante identificar os fatores de risco para nefrolitíase. As doenças inflamatórias intestinais, que incluem a doença de Crohn, representam fator de risco para a formação de cálculos renais, devido à própria doença e ao uso de medicamentos que podem influenciar o metabolismo de substâncias relacionadas à nefrolitíase. No passado, os pacientes com doença de Crohn eram frequentemente submetidos a cirurgia, o que é conhecido por contribuir para a nefrolitíase. Novos medicamentos alteraram o curso clínico da doença de Crohn, cuja incidência aumentou em todo o mundo. Os especialistas devem estar atentos não apenas às complicações dela, mas também as manifestações extraintestinais da doença, que podem afetar dramaticamente a qualidade de vida desses pacientes e levar à insuficiência renal. Sendo assim, é importante rastrear esta população para nefrolitíase.

**Objetivo:**

Determinar a prevalência de nefrolitíase numa população de doentes com doença de Crohn; determinar se medicamentos, cirurgia prévia, localização da doença e atividade clínica são fatores de risco para nefrolitíase; e alertar os especialistas para a importância do rastreio da nefrolitíase (através de métodos simples) para prevenir a doença renal crônica.

**Métodos:**

Foram analisados os prontuários eletrônicos de 93 pacientes com doença de Crohn atendidos entre 2009 e 2010, no Centro de Doenças Inflamatórias Intestinais da Clínica Scope, localizado na cidade Campo Grande. Todos os pacientes foram submetidos à ultrassonografia na primeira consulta médica.

**Resultados:**

Dos 93 pacientes, 37 desenvolveram nefrolitíase em algum momento durante o período do estudo. Os fatores de risco para nefrolitíase foram localização da doença (*P*=0,023) e uso de ciprofloxacino (*P*=0,0001), corticoide (*P*=0,005), imunomoduladores (*P*=0,001) ou metronidazol (*P*=0,0005). O estado cirúrgico, a idade e o sexo não predispuseram à formação de cálculos renais.

**Conclusão:**

Este estudo demonstra a importância do uso de métodos de imagem para triagem de nefrolitíase em pacientes com doença de Crohn, independentemente do estado cirúrgico.

## INTRODUCTION

Nephrolithiasis is a common condition that affects the quality of life and professional activity of patients. The rates of recurrence and urological interventions in patients with nephrolithiasis are high, and nephrolithiasis patients account for approximately 5% of all patients undergoing dialysis. Nephrolithiasis is also a possible cause of terminal renal failure. Therefore, it is important to diagnose nephrolithiasis as early as possible in order to perform a metabolic or urological intervention (lithotripsy). The incidence of nephrolithiasis has increased in recent years, from 3.8% in the 1970s and 1980s to 5.2% in the 1990s. The prevalence is higher among males, and most patients are asymptomatic; therefore, it is important to be on the alert for renal calculi, in order to prevent chronic kidney disease^1^.

Chronic kidney disease is the leading cause of morbidity and surgical interventions in the United Kingdom; factors such as gender, genetic predisposition, obesity, diabetes, and high animal protein intake should be taken into consideration, because they have been reported to increase the risk of nephrolithiasis^2^. 

It has been estimated that approximately 75% of patients with nephrolithiasis present with calcium oxalate stones, which are therefore the most common renal calculi, followed by calcium phosphate stones, uric acid stones, and struvite (ammonium magnesium phosphate) stones; in addition, calcium oxalate stones have been reported to be common in patients with Crohn’s disease^3^. 

One of the most widely used methods for identifying renal calculi is ultrasound, which is due to its ease of use and low cost; some studies have reported that the specificity of ultrasound for the diagnosis of renal calculi can be as high as 95%^4^.

In patients with inflammatory bowel disease, ultrasound is extremely important as a screening method, since the rates of renal calculi in these patients have progressively increased in the last 20 years, from 3% to approximately 8.6%. These rates can be as high as 16% in inflammatory bowel disease patients who have undergone surgery^5^. 

Patients with Crohn’s disease tend to present chronic volume contraction due to fecal water loss (caused by diarrhea, which is common in these patients); this loss leads to decreased urine volume and contributes to nephrolithiasis^6^. It is also known that gastrointestinal disease is accompanied by metabolic changes, such as lithiasis, hypocitraturia, hyperoxaluria, and hyperexcretion of uric acid^4^ ^,^ ^7^. 

In addition to the metabolic factors, the drugs used in the treatment of inflammatory bowel disease (Crohn’s disease and ulcerative colitis) - including sulfasalazine, 5-aminosalicylic acid (5-ASA), metronidazole, ciprofloxacin, and immunomodulators -can be risk factors for the formation of renal calculi. Therefore, specialists should be on the alert for such factors in order to prevent complications of nephrolithiasis associated with the underlying disease. 

The objectives of the present study were as follows to determine the prevalence of nephrolithiasis in a population of patients with Crohn’s disease. Also to evaluate the risk factors for nephrolithiasis and the potential means of reducing those factors, to alert specialists to the importance of screening this condition (through simple methods) in order to prevent chronic kidney disease, to evaluate the risk of developing nephrolithiasis associated with the use of the drugs employed in the treatment of Crohn’s disease, to determine whether the extent, location, and behavior of Crohn’s disease are risk factors for nephrolithiasis and to determine whether gender and having undergone surgery contribute to the formation of renal calculi.

## METHODS

This was a prospective study, conducted by one year. 

The study comprised 93 patients with Crohn’s disease who were treated at the Inflammatory Bowel Disease Center of the Scope Clinic, located in the city of Campo Grande, between 2009 and 2010. All patients underwent ultrasound screening at the first medical appointment. In cases where renal calculi were observed on ultrasound, patients were referred to a nephrologist for diagnostic confirmation, treatment, and follow-up. 

The following risk factors were analyzed for renal calculi: previous surgery; drug use; location of Crohn’s disease; extent of Crohn’s disease; behavior of Crohn’s disease; gender and age. 

In order to stratify the patients, we employed the Vienna classification^4^, which categorizes Crohn’s disease as follows: by age (A) at diagnosis (A1: < 40 yr or A2: ≥40 yr); by behavior (B) of the disease (nonstricturing nonpenetrating, stricturing, or penetrating-B1, B2, or B3, respectively), and by location (L) of the disease (terminal ileum, colon, ileocolon, or upper gastrointestinal tract-L1, L2, L3 or L4, respectively). 

### Statistical analysis

Student’s *t*-test and chi-square test were employed. When the number of patients within a given extract was less than 5, Fisher’s exact test was employed. Analysis of variance were used in the statistical investigation of all data. Values of *P*<0.05 were considered statistically significant. 

The study was approved by the Sao Paulo’s University (USP) ethics committee (182330).

## RESULTS

Of the patients evaluated, 38.7% had renal calculi. Although 52% of the patients were female, gender was not found to be a risk factor for developing renal calculi (*P*=0.934). Patient ages ranged from 31 to 51 yr, and the data showed normal distribution. There were no significant differences between patients with renal calculi and those without in terms of age (*P*=0.680). All of the data were stored in a database in order to facilitate the monitoring of the patients and the analysis of the clinical course of the disease.

As can be seen in Table 1, in terms of the behavior of the disease, patients with B1 disease predominated (66 patients), followed by those with B2 disease (12 patients) and those with B3 disease (11 patients). 

**TABLE 1:** Characteristics of the patients evaluated (n=93).

Age <40, % (95%CI)	40 (31-51)
Female, %	52
CD disease phenotype (behavior), %	
B1	66
B2	12
B2/B3	11
B3	3

CD: Crohn’s disease; B1: nonstricturing nonpenetrating; B2: stricturing; B3: penetrating.

Regarding the location of the disease, 77.5% of the patients had L1 disease, 3.2% had L1/L4 disease, 5.6% had L2 disease, and 14.6% had L3 disease. There were significant differences between Crohn’s disease patients in terms of the location of the disease. The proportion of Crohn’s disease patients with L1 disease was significantly greater than was that of those with L1/L4, L2, or L3 disease (*P*<0.001), whereas that of those with L3 disease was significantly greater than was that of those with L1/L4 or L2 disease (*P*=0.005 and *P*=0.044, respectively). 

These locations were considered to constitute a risk factor for the formation of renal calculi (*P*=0.007). Nephrolithiasis was more common among the patients with L3 disease than among those with L1 disease and those with L2 disease (*P*=0.005 and *P*=0.007, respectively). 

Disease activity was determined by Crohn’s disease activity index (CDAI), which consists of eight items, among which are general well-being and use of antidiarrheals. Patients who presented with a CDAI >250 were classified as having active disease, whereas those who presented with a CDAI <250 were classified as having inactive disease. Neither disease activity nor previous surgery influenced the formation of renal calculi (*P*=0.603 and *P*=0.477, respectively). 

As can be seen in Table 2, the use of drugs such as metronidazole, ciprofloxacin, immunosuppressants, and corticosteroids had a direct influence on the formation of renal calculi. 

**TABLE 2:** Influence that drug used in the treatment of Crohn’s disease had on the formation of renal calculi in the patients evaluated (n=93).

Variable	OR	95%CI	P
Cortisone	NS	NS	NS
Ciprofloxacin	5.2	1.9-14	0.002
Immunomodulator	4.2	1.7-10	0.001
Metronidazole	3.5	1.3-9.8	0.002
Methotrexate	3.8	1.3-11	0.01
Infliximab	NS	NS	NS
Adalimumab	NS	NS	NS

CI: confidence interval; NS: not significant.

## DISCUSSION

Nephrolithiasis is one of the most common preventable diseases of the urinary tract. A new investigative algorithm, recently presented by Hippisley-Cox and Coupland (2010)^8^, is aimed at the timely identification of nephrolithiasis in the general population, in order to prevent chronic kidney disease^2^. The authors call attention to the risk factors for nephrolithiasis and recommend the use of ultrasound as a screening method^9^. 

Among the risk factors for nephrolithiasis are pathologies that are accompanied by diarrhea. Crohn’s disease is a chronic, recurrent disease, and one of its principal clinical characteristics is diarrhea. Although Crohn’s disease can affect any part of the gastrointestinal tract, it most commonly affects the terminal ileum; however, extraintestinal manifestations, such as nephrolithiasis, are common, principally in patients who have undergone surgery^10^. 

The occurrence of nephrolithiasis in patients with Crohn’s disease is quite high (16%), and it is believed that the long-term frequency of nephrolithiasis can be as high as 28% in Crohn’s disease patients in whom more than 100 cm of the ileum have been resected^11^. 

Nephrolithiasis in patients with Crohn’s disease is intimately related to chronic diarrhea, which can be due to bowel resection (generally of the small bowel) or to the underlying disease itself. Numerous studies have shown that the surgical status of Crohn’s disease patients plays an extremely important role in the formation of renal calculi^4^. Calcium is habitually absorbed, through active and passive transport, in the small intestine. Active transport is regulated by vitamin D and its metabolites, whereas passive transport occurs when the concentration of calcium is excessively high, as occurs when the daily intake of calcium is 1-2 g. Therefore, small bowel resection can interfere with the absorption of calcium and lead to hypercalcemia. Hypervitaminosis D can also be caused by the individuals themselves, when the intake of vitamin D is inadvertently high, causing abnormally high systemic levels of calcium and blocking the intestinal absorption of calcium. Patients with inflammatory bowel disease can require calcium supplementation due to osteoporosis, which sometimes accompanies the underlying disease^11^ ^-^ ^13^. In addition, such patients have chronic diarrhea, which can lead to dehydration, decreased urine volume, and volume contraction due to the loss of water and electrolytes in the stool; this leads to urinary supersaturation of substances, such as calcium oxalate, which are not absorbed together with fatty acids^4^ ^,^ ^11^. Hyperoxaluria, which is related to the malabsorption of bile salts and fatty acids, is one of the most common causes of lithiasis and is associated with chronic diarrhea and bowel resection^4^ ^,^ ^11^. Furthermore, the loss of fluids (through diarrhea) reduces urine volume and can lead to metabolic acidosis, which is in turn associated with a reduction in citrate (metabolic acidosis and dehydration) and hyperuricemia, leading to hyperexcretion of uric acid (dehydration). 

Patients who have undergone small bowel resection also develop hyperoxaluria because large quantities of oxalate are absorbed, once oxalate is free in the intestinal lumen; this explains why nephrolithiasis predominantly manifests as calcium oxalate stones in these patients; however, in patients who have undergone colon resection with ileostomy, there is excessive bicarbonate loss, and the formation of uric acid stones is more common^14^ ^-^ ^16^. 

The present study investigated 93 patients. Of these, only 9 had previously undergone any type of surgery. 

In addition, 18 of the patients had undergone ultrasound before the diagnosis of nephrolithiasis, and only five presented with renal calculi, the mean time elapsed between the date of the ultrasound and the diagnosis of nephrolithiasis being 2 yr. Of the 27 patients who had undergone ultrasound and had been diagnosed with nephrolithiasis in the same year, 10 had renal calculi. Of the 93 patients evaluated, 37 presented with renal calculi at some point during the study period. 

Of the 37 patients who presented with renal calculi at some point during the study period, 9 developed hydronephrosis. However, none of those patients required surgical intervention, and the evolution of hydronephrosis was favorable. When we compared Crohn’s disease patients with and without nephrolithiasis, we found that the risk of developing hydronephrosis was significantly higher in the latter (*P*=0.024). Of the 37 patients who presented with renal calculi, seven also developed urinary tract infection. Were found no significant differences between patients with renal calculi and those without in terms of the risk of developing urinary tract infection (*P*=0.101). However, when we analyzed urinary tract infection and hydronephrosis, we observed that the risk of developing hydronephrosis or urinary tract infection was significantly higher in patients with renal calculi than in those without (*P*=0.019). Hydronephrosis has been described as a common manifestation in patients with Crohn’s disease. Ben et al. analyzed renal complications in patients with Crohn’s disease and reported that, of the 312 patients evaluated, 22 had urinary tract infection (cystitis)-for which some required hospitalization-and 4 had hydronephrosis^17^. A similar study conducted in Japan evaluated 41 patients with Crohn’s disease and demonstrated hydronephrosis (diagnosed by ultrasound) in 10. The authors also reported that hydronephrosis is not as uncommon and it is extremely important that patients with Crohn’s disease undergo periodic ultrasound examination^18^. 

Although some studies have reported that renal calculi are more common in males than in females, a study conducted by Semins et al. (2010) showed that being a female, associated with other factors, such as lifestyle and obesity, contributes to nephrolithiasis in patients with Crohn’s disease. In the present study, no influence of gender was found on nephrolithiasis, nor were there any significant differences between patients with renal calculi and those without in terms of age. 

Disease location represented a risk factor for developing nephrolithiasis, and there were significant differences between the proportion of patients with renal calculi and that of those without in terms of the location of the disease. 

The behavior of the disease did not represent a risk factor for developing nephrolithiasis. Likewise, having undergone surgery did not represent a risk factor for developing nephrolithiasis, and no significant differences were observed among the patients with renal calculi between the proportion of those who had undergone surgery and that of those who had not. 

In our study sample, the number of individuals who had undergone surgery was small (n=9). However, this is representative of the changes that have occurred in the clinical course of Crohn’s disease in the last decade, with the advent of new drugs and numerous pharmacological approaches. There has been a drastic decrease in the rates of surgery and an increase in the therapeutic armamentarium employed in the clinical treatment of Crohn’s disease patients, in the active and maintenance phases of the disease^17^ ^-^ ^19^. 

Among the pharmacological approaches to Crohn’s disease, the use of 5-ASA derivatives has been reported as being a risk factor for developing renal calculi and interstitial nephritis^20^. 

In a study conducted in the United Kingdom^20^, it was reported that patients with inflammatory bowel disease who received treatment with 5-ASA were more likely to develop renal calculi than were those who did not. In the present study, was found no significant differences among the patients with renal calculi between the proportion of those who used 5-ASA and that of those who did not (*P*=1.000). 

Drugs such as metronidazole, ciprofloxacin, methotrexate, immunomodulators, corticosteroids, and immunosuppressants (such as azathioprine) are part of the therapeutic armamentarium for the treatment of patients with Crohn’s disease. In the present study, the use of some of these drugs was found to be a risk factor for the formation of renal calculi (Table 2). 

The use of drugs such as quinolones, which are widely used in patients with kidney disease (principally in those with urinary tract infection), has not been reported to be a risk factor for nephrolithiasis. In patients with L2 Crohn’s disease, quinolones can avoid relapse, because, similarly to metronidazole, they act as immunomodulators of the inflammatory response^21^. 

We believe that metronidazole and ciprofloxacin contribute to nephrolithiasis by reducing the population of *Oxalobacter formigenes*, a Gram-negative, anaerobic bacterium that is present in a large proportion of the normal adult population. Although there have been no reports confirming that hypothesis, *O. formigenes* is sensitive to drugs such as clarithromycin and doxycycline. These bacterium catabolize oxalate in the intestine, thereby inducing a decrease in the levels of oxalate in plasma and urine^7^ ^,^ ^22^. 

There have been no reports in the literature stating that immunosuppressants, methotrexate, or anti-tumor necrosis factor agents contribute to the formation of renal calculi in any way. In these present study, the drugs used in the treatment of Crohn’s disease appear to have increased the risk of nephrolithiasis, once these drugs are administered in cases that are more severe and in which the response to the earlier stages of treatment is unfavorable. Therefore, the risk of developing nephrolithiasis is directly related to the underlying disease. All of these effects are shown in Table 2. 

## CONCLUSION

The present study underscores the importance of performing multiple screening tests in patients with Crohn’s disease, principally tests that screen for extraintestinal manifestations, such as renal calculi. The location of the disease and the drugs that are used to treat Crohn’s disease constitute major risk factors for developing nephrolithiasis. Therefore, patients with Crohn’s disease should be screened for renal calculi on a regular basis, in order to prevent diseases that are more severe, such as chronic kidney disease, which is intimately related to nephrolithiasis. 
